# Acceptability of active case finding with a seed-and-recruit model to improve tuberculosis case detection and linkage to treatment in Cambodia: A qualitative study

**DOI:** 10.1371/journal.pone.0210919

**Published:** 2019-07-02

**Authors:** Sovannary Tuot, Alvin Kuo Jing Teo, Danielle Cazabon, Say Sok, Mengieng Ung, Sangky Ly, Sok Chamreun Choub, Siyan Yi

**Affiliations:** 1 KHANA Center for Population Health Research, Phnom Penh, Cambodia; 2 Faculty of Social Science and Humanities, Royal University of Phnom Penh, Phnom Penh, Cambodia; 3 Saw Swee Hock School of Public Health, National University of Singapore and National University Health System, Singapore, Singapore; 4 McGill International TB Centre, McGill University Health Centre, Montréal, Canada; 5 Humanities and Social Studies Education Academic Group, National Institute of Education, Nanyang Technology University, Singapore, Singapore; 6 Center for Global Health Research, Touro University California, Vallejo, the United States of America; University of South Florida, UNITED STATES

## Abstract

**Background:**

With support of the national tuberculosis (TB) program, KHANA (a local non-governmental organization in Cambodia) has implemented an innovative approach using a seed-and-recruit model to actively find TB cases in the community. The model engaged community members including TB survivors as seed and newly diagnosed people with TB as recruiters to recruit presumptive TB cases in their social network in a snowball approach for screening and linkage to treatment. This study aimed to explore the acceptability of the active case finding with the seed-and-recruit model in detecting new TB cases and determine the characteristics of successful seeds.

**Methods:**

This qualitative study was conducted in four provinces (Banteay Meanchey, Kampong Chhnang, Siem Reap, and Takeo) in Cambodia in 2017. Fifty-six in-depth interviews and ten focus group discussions (with a total of 64 participants) were conducted with selected beneficiaries and key stakeholders at different levels to gain insights into the acceptability, strengths, and challenges in implementing the model and the characteristics of successful seeds. Transcripts were coded and content analyses were performed.

**Results:**

The seed-and-recruit active case finding model was generally well-received by the study participants. They saw the benefits of engaging TB survivors and utilizing their social network to find new TB cases in the community. The social embeddedness of the model within the local community was one of the major strengths. The success of the model also hinges on the integration with existing health facilities. Having an extensive social network, being motivated, and having good knowledge about TB were important characteristics of successful seeds. Study participants reported challenges in motivating the presumptive TB cases for screening, logistic capacities, and high workload during the implementation. However, there was a general consensus that the model ought to be expanded.

**Conclusions:**

These findings indicate that the seed-and-recruit model is well-accepted by the beneficiaries and key stakeholders. Further studies are needed to more comprehensively evaluate the impacts and cost-effectiveness of the model for future expansion in Cambodia as well as in other resource-limited settings.

## Introduction

Cambodia is one of the 30 countries in the world with a high burden of tuberculosis (TB) [1]. In 2017, the incidence of all forms of TB was 326 in 100,000 population (95% CI 224–447) [1]. These rates have reduced substantially since 1990, and a similar decline was observed in the TB mortality rate [2,3]. The country has also made notable progress in the fight against TB by achieving a treatment success rate of 94%, one of the highest among the 30 countries with high TB burden [3]. However, the successes are impeded by a significant proportion of undiagnosed cases. Globally, it is estimated that 36% of TB cases were undiagnosed in 2017, and a similar proportion is observed in Cambodia [4–6].

TB disproportionately affects key and vulnerable populations including people living with HIV, household and close contacts of bacteriologically confirmed cases, elderly above the age of 55, people with diabetes, prisoners, migrant workers, the poor, and those living in rural communities [7]. Despite a well-established national TB infrastructure, these key populations still face geographical, economic, social, and biomedical barriers to diagnosis and treatment services [7,8]. In 2016, Ngin and colleagues reported that people with TB received inadequate consultation at both health centers and referral hospitals, and routinely experienced delays in diagnosis and treatment initiation [9]. Furthermore, health center staff, laboratory staff, and village health support groups (a network of community health volunteers that link presumptive TB cases in the community to the health facilities) were found to be insufficiently motivated in the conduct of their work [9].

Traditionally, TB cases are captured and passively notified when they present themselves to a health facility. To further find undiagnosed cases and curb TB transmission, the National Center for Tuberculosis and Leprosy Control (CENAT) has adopted a proactive approach to find cases and promptly link them to care [4,6,10]. Despite the evidence of feasibility and cost-effectiveness of implementing active case finding among urban poor and rural settings in Cambodia [10,11], TB case finding remains a great challenge due to limited resources, geographical barriers, and social stigma. Finding new TB cases remains a great challenge given the stigmatization associated with TB and its highly contagious nature [12–14].

Empirical evidence suggests both efficiency and effectiveness in using a “snowball” approach to engage hard-to-reach key populations for human immunodeficiency virus (HIV) in many countries, including Cambodia [15–19]. However, little is known about the feasibility, acceptability, and effectiveness of this approach in improving the detection of new TB cases. Given the comparable challenges in engaging key populations for HIV and presumptive TB cases in the community, it is a strategy worth exploring.

**Fig 1:**
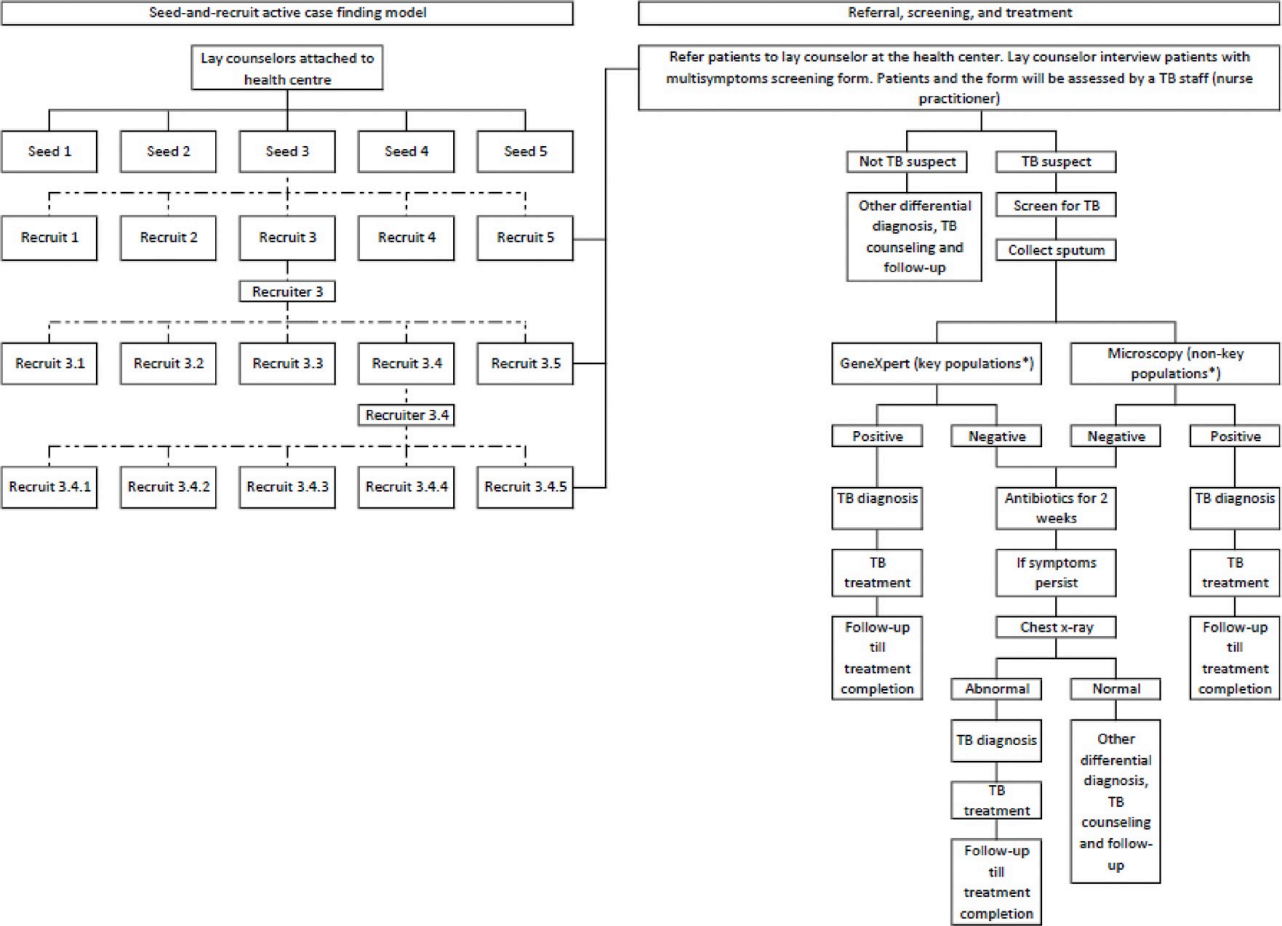
Active case finding with seed-and-recruit model. Dotted lines in the left panel refer to a network that a seed could build potentially. Recruits who meet the criteria of a recruiter will be trained to recruit other people who might have TB in the community. *Key populations: elderly above the age of 55, people with diabetes, people living with HIV, household contacts of TB patients, people who use and inject drugs, prisoners.

To address this knowledge gap, KHANA (a local non-governmental organization in Cambodia) has implemented active case finding with a seed-and-recruit model in four national priority provinces in Cambodia (Fig 1). This model is a system of rapid, targeted low-cost community social mobilization, involving people who themselves had experienced TB (TB survivors), to increase case finding in the provinces. A lay counselor was responsible for two health centers in an operational district. Lay counselors act as focal points at the health center responsible for identifying seeds, conducting pre-screening of presumptive TB cases, and training of potential recruiters. At the initial stage, each lay counselor was tasked to identify five TB survivors per health center as seeds to identify presumptive TB cases (recruits) in their community. These new recruits were screened for TB at the health centers and linked to care if TB was diagnosed (Fig 1). Among the recruits, those who were eligible were trained to become recruiters to find other presumptive TB cases in a snowball approach. Eligibility criteria for lay counselors, seeds, and recruiters are listed in S1 File. This study aims to document the acceptability of the active case finding with the seed-and-recruit model in detecting new TB cases and determine the characteristics of successful seeds.

## Materials and methods

### Ethics statement

This study was approved by the National Ethics Committee for Health Research (N. 238 NECHR) of the Ministry of Health in Cambodia. A letter of support was obtained from each provincial health department of the four provinces where the intervention was implemented. The interviewers explained all relevant study information, and a copy of the information sheet was provided to the participants before data collection started. Due to low literacy and to maintain anonymity, verbal consent was granted by the ethics board in lieu of written consent.

### Study design and settings

This qualitative study was conducted from November to December 2017 in four provinces, where the seed-and-recruit model was implemented–Banteay Meanchey, Kampong Chhnang, Siem Reap, and Takeo. The Consolidated Criteria for Reporting Qualitative Research (COREQ) checklist was followed (see S2 File).

### Sampling and participant recruitment

We conducted 56 in-depth interviews (IDIs) with lay counselors, TB program staff at health centers and referral hospitals (TB program officers and clinical staff), village health volunteer groups, seeds, field staff (field officers overseeing project implementation in the operational district), community-based directly observed treatment short course (C-DOTS) volunteers (volunteers responsible for the monitoring and follow-up of TB treatment in the community) and people with TB. We also conducted ten focus group discussions (FGDs) with four groups of participants–people living with HIV, people with diabetes, elderly aged 55 and above, and representatives of the general population (those aged between 18 to 54 years, including TB survivors). A guide for IDIs and FGDs is attached as S3 File. A stratified purposive sampling method was employed to recruit the study participants. Potential study participants (people with TB, presumptive TB cases, and members of key populations) and representatives from the provincial health departments, operational districts, and health facilities were invited either in-person or via the telephone by the research team. All potential participants aged 18 and above were eligible. All presumptive TB cases and those diagnosed with TB recruited for this study were referrals made to the health facilities via the seed-and-recruit model.

### Data collection

Interviews were performed by 10 data collectors (five Cambodian men and five Cambodian women, aged below 30 years) who had experiences in qualitative research and were trained and closely supervised by the principal investigators. Information on the study and its objectives were provided verbally to potential participants. Interviews were arranged with those who agreed to partake at a time and location of convenience that included participants’ houses, pagodas, health centers, and offices. The IDIs and FGDs took between 30 minutes to one hour and were audio-recorded. A token of appreciation (USD$5) was given to participants at the end of the interview.

The IDIs and FGDs were conducted in private places using a semi-structured guide in Khmer. The guide was comprised of broad themes and aimed to understand the process, feasibility, acceptability, and strengths of the model in TB case detection and linkage to care. We also sought to understand the challenges faced by seeds in implementing this intervention and in identifying the characteristics of seeds that may potentially contribute to the success of this intervention. The guide was pilot-tested before the implementation. Individuals who participated in the pilot test were excluded from the main study.

### Data management and analyses

All data were transcribed verbatim into Khmer by members of the research team. Personal information and identifiers were removed from the final transcripts and field notes to ensure anonymity and confidentiality. The co-investigator with an MA in Health Social Science (TS) was the sole coder in this study. Transcripts were coded in NVIVO 11 (QSR International). The initial themes based on the semi-structured guide were used to develop a codebook. We also performed content analyses to identify emerging categories, themes, and common and divergent patterns pertinent to the objectives of the study. We described socio-demographic information of the study participants using STATA 14 (StataCorp, LP, Texas, USA).

## Results

### Characteristics of study participants

In total, we conducted 56 IDIs and 10 FGDs (with 64 participants). Of the 10 FGDs, four FGDs were conducted with elderly above the age of 55 and members of the general population (e.g., people below the age of 55, TB survivors). Two separate FGDs were conducted with people with diabetes and people living with HIV. Of the 56 IDIs, more than half of the participants were male. The median age was 51 (ranging from 17 to 88) years. One-third of the FGD participants were male, and the median age was 54 (ranging from 17 to 78) years. Characteristics of the participants in IDIs and FGDs are summarized in Table 1.

**Table 1:** Characteristics of participants in in-depth interviews and focus group discussions.

Demographic variables	*n* (%)	
Sex		
Male	51 (42.5)	
Female	69 (57.5)	
Age, years, median (range)*	53 (17–88)	
Level of education*		
No education	22 (22.4)	
Primary	46 (46.9)	
Secondary	30 (30.6)	
	IDI, *n* (%)	FGD, *n* (%)
Total number of IDIs/FGDs conducted	56	10^†^
Elderly (age 55 and above)	4 (7.1)	4 (40.0)
People with diabetes	3 (5.4)	1 (10.0)
People living with HIV	5 (8.9)	1 (10.0)
Representatives of the general population (those aged between 15 to 54 years, including TB survivors)	5 (8.9)	4 (40.4)
PHD and OD	7 (12.5)	
Referral hospital and health center	8 (14.3)	
Field staff	4 (7.1)	
C-DOTS volunteer	3 (5.4)	
Lay counselor	7 (12.5)	
Seeds, recruiters, and the VHSG	10 (17.9)	

*IDI*, *in-depth interviews; FGD*, *focus group discussion*, *PHD*, *provincial health department; OD*, *operational district; C-DOTS*, *community-based directly observed treatment short course; VHSG*, *village health support groups*.

^*^Exclude missing values (n = 22).

^*†*^*10 FGD with a total of 64 participants*.

### Acceptability of the seed-and-recruit model

In general, the study participants found the seed-and-recruit model helpful for both the beneficiaries and the community. They appreciated that people who are sick would be screened and treated on time through this model. The project staff, lay counselors, seeds, and health care providers could see a lot of benefits of engaging TB survivors (and the snowball approach, more broadly) to recruit potential people with TB. The seeds could easily identify people with TB symptoms for screening and encourage them to seek care. Hence, they perceived that the model must be expanded to halt transmission and eliminate TB from the community.

*“It is acceptable because I can see that they (seeds and recruiters) have been active in the search and thus the number of TB cases will be decreasing in the future”* (IDI with a female health center staff member from Kampong Chhnang)

### Benefits and satisfaction with the seed-and-recruit model

The seed-and-recruit model mobilizes communities to seek people with TB symptoms and empowers them to be conscious about their health.

*“I think the model is good for me*. *Sometimes we forget to take care of our health*, *and they are a good reminder for us to check up our health*.*”* (IDI with a woman living with HIV from Banteay Meanchey)

Many respondents could see the strengths in the model. The study participants expressed that the model encouraged early diagnosis and treatment. Patients were also guided during the screening and treatment process by the seeds at no cost.

*“A positive thing (about the project) is that our villagers are alerted earlier before TB gets worse*. *It is good that they come to search for us and guide us through the screening and treatment and to educate us too*. *We do not need to pay a cent*.*”* (FGD with a female elderly from Banteay Meanchey).

The ability of the model to identify more TB cases through trained seeds and recruiters were identified as one of the main strengths of the model.

*‘Mobilizing well-trained seeds (and recruiters)*, *who can identify more TB cases and send them to take the test at a health facility*, *is one of the strengths of the model*.*”* (IDI with a male member of the village health support group from Banteay Meanchey)

This peer-driven model was a ground-up and community-based intervention. Study participants expressed that this model played a pivotal role in behavior change through skills building, peer influence, motivation, and knowledge sharing. An intervention as such, nested in the community, was perceived to be sustainable.

*‘Social embeddedness of the seeds and recruiters in the local community and hence the ability to share knowledge and experience with the potential recruits*, *leading to sustainability of the intervention*.*’* (IDI with a male health center staff member from Takeo)

Study participants expressed that the model can be scaled up and expanded nationwide due to the potential impact on TB case detection and cost-effectiveness of the intervention. The project also met the national program’s mission to control TB in the country.

*“I think it can be scaled up because the model is at low cost*, *and when we search for the TB patients everywhere*, *the intervention can be effective and TB burden will be reduced*.*”* (IDI with a female community DOTS volunteer from Siem Reap)

*“The special feature of this model is that our seeds are TB survivors*, *and hence they know the symptoms well and what to talk to potential recruits*. *This approach can produce high yields with little budget”* (IDI with a male health center staff member from Takeo)

*“It is scalable to the national level*. *If it is properly executed*, *we are able to find many cases*. *This also aligns with the national guidance/principles*, *which want health centers to look for new cases”* (IDI with a female referral hospital staff member from Siem Reap)

### Integration of the model into the Cambodian health system

The integration of the model into the existing public health care systems was well-received by the study participants. The health centers were well-maintained and located conveniently close to their residences. They also commended the health center and project staff for their professionalism.

*“Good cooperation with health facilities*, *health administrators*, *and local authorities*.*”* (IDI with a female referral hospital staff from Siem Reap)

*“Strong and genuine commitment from (many) lay counselors and seeds and recruiters in their work and the cause*.*”* (IDI with a female referral hospital staff from Takeo)

Field staff, lay counselors, seeds, recruiters as well as health center, operational district, and provincial staff agreed that having adequate TB screening facilities and health personnel at the health facilities are crucial for the success of the model. Generally, there are no shortage of staff and TB screening facilities at most locations.

*“There is seemingly no staff shortage*.*”* (IDI with a male operational district staff from Banteay Meanchey)

Nonetheless, a concern about health facilities’ inability to handle the volume of workload if the intervention is scaled up was raised and a systematic solution ought to be explored.

*“In the future*, *when we can find more cases*, *the challenge could be that we could not handle the cases as we have limited number of staff*. *Maybe in that case*, *we will need to have a proper appointment system with the recruits*.*”* (IDI with a female health center staff member from Kampong Chhnang)

There were also instances of limitations in the screening capacities in some health centers. Some participants stated that the limited number of staff and diagnostic infrastructures at the health facilities occasionally prevented timely management and process of samples, resulting in a delay of diagnosis. In the matter of consumables outage, study participants concurred that they generally did not face major issues. If shortages were to occur, communications with other partners were swift to resolve the problem.

*There is only one GeneXpert machine at the health facility*, *and it is used to conduct sputum test from our project as well as from another NGO and the health facility itself*. *Therefore*, *sometimes they cannot handle all of them on time*. *In one day*, *at most*, *it can handle 16–20 bottles*. *Sometimes we need to wait for half a month to get the result*.*”* (IDI with a male lay counselor from Siem Reap)

*“Like ink for the GeneXpert machine*, *occasionally they (health facilities) may run out of stock*. *However*, *we contacted the operational district straight away*, *and it was immediately resolved*.*”* (IDI with a male local project officer from Banteay Meanchey)

### Challenges in implementing the seed-and-recruit model: presumptive TB cases lacked motivation to participate

Nevertheless, difficulties in motivating potential people with TB symptoms to seek screening and treatment services was expressed as one of the major challenges in the model implementation. It was attributed to several issues–personal reasons and competing priorities (work, household chores), denial, and misconception regarding TB. Furthermore, additional trips to the referral hospital for further testing were not well-received by potential patients due to the distance between the referral hospital and their residence.

*“Some people are very difficult to talk to*. *Some people are ignorant and stubborn*. *People aged 70 and above are hard to talk to because they think that no one in their families has ever had TB*.*”* (IDI with a female seed from Banteay Meanchey)

*“Lack of cooperation from potential recruits in further recruitment (e*.*g*. *because of old age*, *busyness with making a living or tending grandchildren) is the main challenge in the model implementation*.*”* (IDI with a male field staff member from Banteay Meanchey)

*“When we meet and give them the coupons*, *they are not interested in them*. *Some are not interested in making use of the coupons*.*”* (IDI with a male seed from Kampong Chhnang)

Study participants highlighted TB-related stigma and discrimination as a barrier to health-care seeking for TB diagnosis. Presumptive TB cases preferred not to discuss TB issues due to shame and fear of being ostracized by others in their community. Therefore, presumptive TB cases were more inclined towards self-treatment than seeking formal health care. The reluctance to come forward for TB diagnosis and treatment was also perceived as a barrier for the seed-and-recruit model to be effectively implemented.

*“They are afraid of being discriminated by others*, *so they treat themselves*.*”* (IDI with male referral hospital staff member from Siem Reap)

*“They are afraid that after the testing*, *when it is a positive result*, *people would stop coming to eat their stuff (patronize their food stall/business)*, *and other people may discriminate against them*. *And one more thing is that*, *for some people*, *we convinced them to come and already did the testing (for TB)*, *but they told us not to enter their house*, *contact them*, *or let other people know because they want to stay hidden and do not want anyone to know about that*.*”* (IDI with male field staff from Takeo)

### Challenges in implementing the seed-and-recruit model: lay counselors and seeds lacked motivation to perform duties

Some lay counselors and seeds reflected that the case-by-case incentivization scheme is not enticing for seeds/recruiters to perform to their full potential.

*“Why don’t they provide us with regular incentives*? *Why are we provided with incentives only when we can find a case*? *When I travel to educate other people*, *I also need to spend time on the work*.*”* (IDI with a female community DOTS volunteer from Siem Reap)

Some local project staff indicated the difficulty in recruiting and maintaining lay counselors due to workload and the lack of incentives. Some study participants expressed that it was challenging for one lay counselor to manage patients in two health centers well and evenly. There was a tendency for a lay counselor to oversee the health centers in his/her residential area more closely and neglect the other one.

*“The work is quite a lot*.*”* (IDI with a male lay counselor from Banteay Meanchey)

*“The incentives are quite little too*.*”* (IDI with a male lay counselor from Kampong Chhnang)

### Challenges in implementing the seed-and-recruit model: lay counselors, seeds, and recruiters insufficiently trained

The study participants indicated that the training is not comprehensive enough for lay counselors, seeds, and recruiters to perform their duties efficiently, given the depth and length of the training sessions. Some participants expressed that the training duration was too short. Hence, it was difficult for the attendees to get a good grasp of the model.

*“Two-day training for people like us would be okay*, *but they [lay counselors and seeds] cannot acquire it in just two days–it is very difficult for them*. *It needs 3–5 days to discuss every theme in any detail*.*”* (IDI with a male operational district staff from Banteay Meanchey)

Apart from the training duration, many other respondents emphasized that the training coverage and depth must be improved and suggested some other areas in which further training will be needed.

*“Some of the selected lay counselors in each OD were TB survivors*, *but they seldom went through training in counseling*. *Their knowledge of TB is limited*, *and their inter-personal communication ability can be limited*. *These require further training*. *Two training areas that may be considered are*: *knowledge and skills to handle (their work within) the project itself and counseling capacity/skills including communication skills to improve their relationship with people with TB (and potential recruits)*, *the local authorities*, *and communities*. *The other thing is to improve their capacity to recruit good seeds*.*”* (IDI with a female field staff from Siem Reap)

### Characteristics of successful seeds

The ability of good seeds in finding new TB cases and mobilizing potential recruits to take the screening test varied from seed to seed. Nevertheless, their achievements in mobilizing testing and yielding positive results were encouraging. Successful seeds/recruiters were driven by both intrinsic and extrinsic motivation (Table 2). Many lay counselors, seeds, and recruiters were motivated by their wish to eliminate TB from their communities, terminate future transmission, and be socially responsible. Incentives, new skills, and knowledge acquisition were also mentioned as reasons to get involved in the project.

*“I want to eliminate the transmission of TB to other people*, *including children*. *I love this work because I used to see the suffering of my mother who went through a hard time when she was seriously ill (i*.*e*., *her lung was destroyed)*, *and I do not want this to happen to other people*.*”* (IDI with a female seed from Siem Reap)

**Table 2:** Characteristics of successful seeds in recruiting people at higher risks and producing high-yield TB screening.

Characteristics of successful seeds
Have time, energy, and genuine commitment to the job and for the cause
Have good basic knowledge of TB (symptoms, care and support, treatment) or willing to receive relevant training on TB
Able to read, write, and communicate
Have good communication skills (able to explain to potential recruits in a friendly and helpful manner)
Have a good social network in the community
Have personal means of transportation

In summary, the following characteristics of successful seeds could be inferred from the study participants that could be emulated in implementing the model in the future.

## Discussion

Overall, the seed-and-recruit model that links community people to TB screening and treatment is deemed to be feasible and acceptable by the beneficiaries and key stakeholders consulted in this study. The appropriateness of applying the seed-and-recruit model in TB case detection and linkage to treatment is similar to the findings in studies that examined its application in HIV programs in Cambodia and elsewhere. In a study by Pitter and colleagues, a peer-driven intervention (PDI) to reach HIV key populations (similar to the seed-and-recruit model) was found to be more effective (yield of HIV positive cases) compared to the same time period in the previous year, and it was positively received by the target populations and program implementers [20]. The study also concluded that the cost per HIV case detected in the intervention arm was three times lower than that of the comparator approach (outreach health counseling and treatment) [20]. Another study by Yi and colleagues on the use of the PDI program to recruit new HIV cases among transgender women also demonstrated the successful application of the approach in Cambodia [19,21].

The effectiveness of community-driven interventions to improve TB diagnosis and care has also been demonstrated in other resource-limited settings. In Myanmar, a community intervention was shown to increase case notification rates compared to household and neighborhood interventions [22]. The involvement of community volunteers in TB control was also found to increase the yield of diagnosis by 12% and reduce diagnostic delay in India and Nepal, respectively [23–25]. A patient-led active case finding strategy implemented in the Democratic Republic of Congo (DRC) reported an increase in TB diagnosis, and the intervention was highly acceptable by the communities, health facilities, and patient leaders. In a separate study in the DRC, a peer education intervention was found to increase TB case notification rates two-fold compared to controls, despite being conducted in conflict-affected settings [26]. Among people living with HIV in Nepal, a peer-led TB case finding intervention resulted in a high yield of TB diagnoses [27]. Besides improvement in TB case notification rates, the peer-led intervention was also shown to improve knowledge on TB and prevention in a study in China [28]. Regarding acceptability of the intervention, respondents found a peer-led intervention to be beneficial and adequate and suggested future expansion [28], concurring with the findings of our study.

An operational research study on the PDI project in HIV conducted by KHANA highlighted challenges in the program implementation such as seed/recruiter drop-out, hard-to-reach key populations, the unattractiveness of the approach (or incentives) to the seeds and recruiters, loss to follow-up, and time constraints for key populations to visit health facilities [29]. While some of these challenges may be specific to the HIV program, our study found similar constraints, and this ought to be critically addressed through education, communication, and sustainable incentivization scheme should the intervention be scaled up nationwide. Efforts were also made to identify the characteristics of good seeds. In the scale-up phase, these findings should be incorporated into the training materials of project staff to identify good seeds efficiently. Coupled with structured and continuous training and monitoring, networks of competent seeds will be instrumental to the success of the project in the future.

Based on the findings from this study, we recommend the following improvements to enhance the intervention for nationwide expansion. First, future programs should provide more logistical support (e.g., stationeries and consumables) for lay counselors, seeds, and recruiters to perform their duties. Support and encouragement in the form of supervision and opportunities to troubleshoot are required to ensure that staff personal health and wellbeing is taken care of, and project implementation is on-track. A more comprehensive training program that includes TB knowledge, interpersonal, administrative, and management skills must be planned for the project to succeed. Furthermore, there is a need to devise a sustainable financial support scheme to recruit and motivate lay counselors, seeds, and recruiters. Second, there is a need for the seed-and-recruit programming to strengthen engagement with relevant stakeholders at all levels to ensure seamless integration into Cambodian health systems and that they are well-informed regarding the project. Their involvement is crucial to scale-up and sustain this intervention in the long run. Finally, there is also a need to devise a support plan and sustainable financial support scheme for health center and referral hospital staff for their services in this project. Finally, public education and sensitization on TB needs to be scaled-up and continued to mitigate stigma. More importantly, the TB community ought to come together collectively to affect practice and policy change to reduce TB-related stigma and discrimination.

This study has several limitations. First, the applicability of the qualitative findings is limited to the health centers included in the pilot phase. Nevertheless, we believe that standpoints that we have collected and synthesized in this study are pivotal to improve the intervention for future scale-up. Second, this study has not demonstrated the efficacy of this approach and further research is required to establish the effectiveness of this model in increasing TB case notifications. Lastly, we were not able to rigorously assess the reliability of the codes due to the use of single coder in this study. However, we strived to mitigate this shortcoming through thorough discussions of the analysis plan and the findings with the research team.

## Conclusions

The seed-and-recruits model is well accepted by the beneficiaries and key stakeholders. The success of this model is anchored to the participatory nature of its design and implementation, the bottom-up driven community empowerment approach, and strong commitment from key stakeholders. We recommend that the scale-up of this intervention integrates other health and social services to the model; continues to support, train, and incentivize lay counselors, seeds, and recruiters; and strengthens engagement with stakeholders at all levels. It is imperative that the project is fine-tuned and subsequently scaled-up countrywide to complement the national efforts to end TB by 2035.

## Supporting information

S1 File Eligibility criteria for lay counselors and seeds and recruiters. (DOCX)

S2 File COREQ checklist for interviews and focus group discussions. (PDF)

S3 File Guide for in-depth interviews and focus group discussions. (DOCX)
