# Supplementary material for: Acceptability of active case finding with a seed-and-recruit model to improve tuberculosis case detection and linkage to treatment in Cambodia: A qualitative study
Source: PLoS One. 2019 Jul 2;14(7):e0210919. doi: 10.1371/journal.pone.0210919 (PMC6605634; doi:10.1371/journal.pone.0210919)
Supplement: S1 File — (DOCX) [file pone.0210919.s001.docx]

**S1 File. Eligibility criteria for lay counselors and seeds and recruiters**

**Eligibility criteria for lay counselors**

1. Able to read and write
2. Residing in the community where work is carried out
3. Have had TB and been cured
4. Has good relationship with the community authorities and health centers
5. Has own transport

**Eligibility criteria for seeds and recruiters**

1. Newly diagnosed TB patients including those undergoing treatment
   1. Know TB symptoms
   2. Volunteers to find other presumptive TB in the community
2. Non-TB patients but belonging to these population groups – family of people living with TB, people with diabetes, and people living with HIV
   1. Know TB symptoms
   2. Know 5 or more presumptive TB in their network
