# Supplementary material for: Acceptability of active case finding with a seed-and-recruit model to improve tuberculosis case detection and linkage to treatment in Cambodia: A qualitative study
Source: PLoS One. 2019 Jul 2;14(7):e0210919. doi: 10.1371/journal.pone.0210919 (PMC6605634; doi:10.1371/journal.pone.0210919)
Supplement: S3 File — (DOCX) [file pone.0210919.s003.docx]

**Supplementary File 2– Guide for In-depth Interviews and Focus Group Discussions**

**Guide for In-depth Interviews with Seeds and Lay Counselors**

1. How old are you?
2. What is your gender?
3. If seed, how long have you been diagnosed with TB?
4. Have you been cured from TB? How long have you been cured?
5. How long have you become a seed/lay counselor?
6. Why did you decide to become a seed/lay counselor?
7. What do you as a seed/lay counselor?
8. How many recruits/seeds have you had so far?
9. What did you do prior to joining this project?
10. Where did you usual find the people to become recruit/seeds?
11. Could you please describe how you found the last person to become recruit/seed? Particular where did you meet? How did you approach him/her? What did you talk about before giving them coupons or refer them to get TB screening?
12. Did you experience any problems or difficulties in recruiting this person (e.g., why do you think they may have hesitated, or what do you think encouraged them to join)?
13. How did you ensure that the persons whom you identified as a seed or recruit were truly at risk of TB infection?
14. In this intervention, do you think active case finding using “seeds and recruits” is acceptable by seeds? Why or why not? Another option: We are using this seed and recruit method to find more people who may have TB and refer them to the clinic. What do you think about this strategy (e.g., do you like it, or do you find it is unacceptable)?
15. In your opinion, what makes a seed a super seed (those who could recruit more TB suspects with better TB screening yield)?
16. Have there been any challenges you have particularly faced that you would like to share with us? (Hints: coordination, staffing, financial incentive…)
17. What kind of pre-test information did you provided to your seeds/recruit before taking the TB screening?
18. How many TB suspects have you found since the project started?
19. How many have you referred for TB screening?
20. How did you refer? Were there any cases who refused TB screening?
21. How many were screened TB positive?
22. Did you provide follow-up support for enrolment in TB treatment in case a test result was positive? What did you tell them?
23. Did you refer all TB positive cases for treatment? If not, why?
24. How did you refer them?
25. Any challenges in the referrals?
26. Have you experienced any differences in how men and women respond to this strategy?
27. What kind of questions did your seeds/recruits usually ask you before they accepted the TB screening and treatment? What did you usually answer them?
28. Did you find the seeds/recruits who changed their mind to not continue after having received a coupon? Did you ask them for reasons? If yes, what were the reasons?
29. Do you feel under pressure sometimes to reach a certain number of seeds/recruits? Could you please describe it? How you overcome the pressure?
30. How do you feel about having to recruit seeds/recruits?
31. Do you think that this intervention can be scaled up to the national level? What makes you think in that way?
32. Based on your personal experience in this project, is there any recommendations you would like to make in order to improve the case detection? (Hints: recommendation to other persons with TB, project staff, national program and donor agencies.

**Guide for In-depth Interviews with Health Providers**

1. What is your main role related to this project? How long have you been involved in this project?
2. In general, do you think the uptake of TB screening could be increased? Why or why not?
3. Could you please tell us some reasons, why some people with TB symptoms may not go for TB screening? What are possible resolutions that may encourage them to go for TB screening?
4. Do you think the lack of materials and staff for TB screening is an important issue in your area? Why and why not?
5. Do you remember who were involved in the implementation of active case finding using snowball approach? How was this done?
6. Do you think training provided by KHANA for people involved in this project is sufficient? What do you think should be considered to improve the project? (Hints: further training, incentive, technical support, etc.)
7. Could you tell us the strengths and weaknesses of this model compared with other TB interventions that you are aware of? (Hints: C-DOTS or ‘expanded case finding’ or semi-active case findings or ‘active case finding’ without “seeds and recruits” model etc…)
8. Is this intervention, active case finding using “seeds and recruits,” acceptable? (Hints: by health providers, seeds, recruits, community people, local authorities, etc.). Why or why not?
9. In your opinion, what makes a seed a super seed (who could recruit more TB suspects with better TB screening yield)?
10. Are there any challenges you particularly faced that you would like to share with us? (Hints: coordination, staffing, financial incentive…)
11. Do you think that this intervention can be scaled up to the national level? What makes you think that way?
12. Based on your personal experience in this project, is there any recommendations you would like to make in order to improve the case detection? (Hints: recommendation to other persons with TB or TB suspects, project staff, national programs and donor agencies, etc.

**Guide for In-depth Interviews and Focus Group Discussions with Beneficiaries**

1. Are you a member of the following target groups?
   1. People living with HIV
   2. 55 years of age and older
   3. Diabetics
   4. Non-KP
2. How old are you?
3. What is your gender?
4. What level of formal education have you completed?
5. What is your main occupation (main source of income)?
6. How long (in months) since the last time you were screened for TB?
7. Where did you meet the seeds?
8. Before taking TB screening, what did the seeds tell you? What did they ask you? (Hints: TB symptoms, importance of the treatment, treatment availability, their experiences in TB screening and treatment, etc.)
9. Have you ever had TB screening before having met with the lay counselor? If yes how long ago? If no what were the reasons that you did not?
10. How do you feel about the experience of receiving support for a TB screening from lay the counselor? What did you expect from them and you did not get?
11. What motivates you to become a recruiter? How many people have you recruited for TB screening since you started working for this project?
12. What do you see as an advantage and a disadvantage of using snowball approach?
13. Is this intervention, active case finding using “seeds and recruits,” acceptable to you? Why or why not?
14. What kind of information you receive before getting TB screening?
15. Did you receive information in relation to the needs, treatment enrolment, and follow up? What did they tell you? What information you wanted to know and were not provided?
16. Did you ask any question to the seed before you volunteered to be a recruit? What did you ask? Did the seed provide you sufficient and clear answers to your questions?
17. At which location was the TB screening conducted by the health center/lay counselor? Did you feel comfortable about this setting? If not why not? What else did you expect?
18. After receiving the TB screening result, what was the lay counselor talk about with you? Were they clear to you? What did you expect to hear more?
19. Do you think that TB can be cured?
20. Based on your experience being a recruit/recruiter, is there any recommendation you would like to make in order to improve the project? (Hints: recommendation to other people with TB, project staff, national program and donor agencies)
